# Isolation and Characterization of Microsatellite Markers for *Cotinus coggygria* Scop. (Anacardiaceae) by 454 Pyrosequencing

**DOI:** 10.3390/molecules19033813

**Published:** 2014-03-24

**Authors:** Wei Wang, Zhuo Li, Yong Li

**Affiliations:** 1 College of Forestry, Henan Agricultural University, Zhengzhou 450002, China; 2 College of Nature Conservation, Beijing Forestry University, Beijing 100083, China

**Keywords:** microsatellite markers, genetic diversity, 454 sequencing, population structure, *Cotinus coggygria*

## Abstract

*Cotinus coggygria* Scop. (Anacardiaceae) is a deciduous shrub or small tree that is native to a large area covering from southern Europe, east across central Asia, and the Himalayas in northern China. Shotgun 454 pyrosequencing was used to develop microsatellite markers from the genome of *C. coggygra.* In this study, 349 microsatellite loci were identified from 40,074 individual sequence reads produced by one-sixteenth run, and primer pairs were designed for these loci. To test the primer amplification efficiency, 50 microsatellite primer pairs were tested across 12 individuals from two *C. coggygria* populations (Wuzhi Mountain: 36°30'N, 113°39'E; Tianlong Mountain: 37°42'N, 112°26'E). Among the 50 tested primer pairs, eight were found to be polymorphic. The average allele number of the microsatellites was 3.5 per locus, with a range from two to five. The inbreeding coefficient ranged from −0.478 to 0.222. The observed and expected heterozygosities varied from 0.167 to 0.750 and from 0.163 to 0.743, respectively. This set of markers is potentially useful for assessing the genetic diversity, as well as for understanding the population structure and phylogeographical and landscape genetic patterns, of *C. coggygria*.

## 1. Introduction

*Cotinus coggygria* Scop. (Anacardiaceae) is a deciduous shrub or small tree that is native to a large area that covers from southern Europe, east across central Asia, and the Himalayas in northern China. In China, this species grows in the area between 32°30' to 42°30' and 103°30' to 124°10' with elevations of 330 m to 2,400 m above sea level [1]. This species grows well under semi-shade and direct sun, and prefers medium levels of water. *C. coggygria* is commonly grown as an ornamental plant and is popularly known for its red autumnal leaves. Previous studies have mainly focused on its ornamental characteristics [2,3], medicinal value [4,5] and cultivation physiology [6,7]. However, the genetic diversity and population structure of the wild population has been rarely explored. Thus, developing a set of more reliable and informative molecular markers to aid in assessing the genetic diversity and the population structure, as well as investigating the phylogeographical and landscape genetic patterns of *C. coggygria*, is essential.

Microsatellites or simple sequence repeats (SSRs) have certain advantages, such as reproducibility, abundant polymorphism, and co-dominant inheritance [8]. Therefore, SSR markers are frequently used to survey population genetics, molecular ecology, and marker-assisted selection studies [9,10,11]. However, traditional methods for isolating SSR markers in non-model organisms involve the construction of the enrichment libraries through magnetic beads and the subsequent positive clone’s verification by polymerase chain reactions (PCRs) [12,13]. These methods are laborious and difficult to use. With technological advances, such as next generation sequencing [14,15,16], the emerging sequencing platforms now allows us to quickly achieve a large number of SSR markers [17,18]. In this study, we utilized shotgun 454 pyrosequencing technology to isolate SSR markers from the *C. coggygria* genome.

## 2. Results and Discussion

In total, 40,074 individual sequence reads with an average length of 422 bp were obtained. Of these, 349 sequences were found to contain simple sequence repeats and deposited in GenBank (KJ398946 - KJ399294). We randomly selected 50 microsatellite primer pairs among the identified SSR loci to test the primer amplification efficiency. Fifteen primer sets were abandoned because of the amplification of multiple bands or unsuccessful amplification of target fragments, whereas the remaining 35 primer pairs were tested for polymorphisms across 12 individuals from two *C. coggygria* populations. Of the 35 tested markers, eight primer pairs yielded polymorphic amplification products (Table 1). The genetic diversity parameters in each population are presented in Table 2. The number of alleles detected at each locus ranged from two (Cc026) to five (Cc025 and Cc047), with an average number of 3.5 per locus. The observed and expected heterozygosities varied from 0.167 (Cc040) to 0.750 (Cc032) and from 0.163 (Cc040) to 0.743 (Cc047), respectively. No locus deviated significantly from Hardy–Weinberg equilibrium (HWE). The inbreeding coefficient ranged from −0.478 (Cc032) to 0.222 (Cc047). Six of eight loci have negative *F*_IS_ values (all *P* > 0.05), which indicate a slight excess of heterozygotes. Genarally, heterozygote excess is caused by small reproductive population size, over dominance, negative assortative mating, or asexual reproduction [19]. As a common species, *C. coggyria* has a large population size in each population. Meanwhile, *C. coggyria* in the wild usually propagates through use of seeds instead of asexual reproduction*.* Thus, the most likely reason for heterozygote excess of *C. coggyria* was inbreeding depression or self-incompatibility. Further experimental verification is needed to test this hypothesis. Significant genotypic disequilibrium was not detected in any pair of loci, indicating there was no significant allelic association between the markers. This is the first set of microsatellite markers developed for this genus and therefore, will be useful for assessing the genetic diversity, understanding the population structure, and phylogeographical and landscape genetic patterns of *C. coggygria*.

## 3. Experimental Section

### 3.1. Isolation of Microsatellite Markers

Total genomic DNA was isolated from *C. coggygria* (dried leaves in silica gel) using plant genomic DNA kit (Tiangen Biotech, Beijing, China) according to the manufacturer's protocol. Approximately 1 μg of genomic DNA was used to generate a shotgun library following the GS-FLX+ library preparation protocol**,** and then sequenced on 454 GS-FLX+ System at Personalbio (Shanghai, China). One sixteenth run was performed, and a total of 40,074 reads with an average length of 422 bp were identified. Microsatellites and microsatellite-flanking PCR primer sequences were searched by Auto-Primer [20]. Parameters were designed for identifying di-, tri-, tetra-, penta-, and hexanucleotide motifs with a minimum of five repeats. Primer design parameters were set as follows: length range, 18–24 nucleotides with 20 as optimum; PCR product size range, 100–500 bp; melting temperature, 55–65 °C; GC content, 40%–60%, with 50% as optimum; and GC clamps, no more than 3 G’s or C’s in the last 5 bases at the 3' end of the primers. The Auto-Primer proposed 349 PCR primer sets for microsatellite loci.

### 3.2. PCR Amplification and Genotyping

Two wild population samples from the Wuzhi Mountain (Hebei Province: 36°30'N, 113°39'E) and the Tianlong Mountain (Shanxi Province: 37°42'N, 112°26'E) were used for polymorphism detection. PCRs were carried out in a PTC-200 thermal cycler (MJ Research, Watertown*,* MA, USA) using in a total volume of 30 µL containing 30 ng of genomic DNA, 0.2 mmol/L of each dNTP, 0.3 µmol/L of each primer, 3 µL a 10× polymerisation buffer (manufacturer), and 1 unit of Taq polymerase (Takara, Dalian, Liaoning, China). The PCR protocols included initial denaturation for 5 min at 95 °C, followed by 35 cycles of denaturation for 40 s at 94 °C, annealing for 45 s at locus-specific annealing temperature (Table 1), and extension for 50 s at 72 °C, with a final extension step of 8 min at 72 °C. Five µL of PCR products was mixed with 1 µL 6× loading buffer, then was electrophoresed on 8% native polyacrylamide gel and visualized using silver staining. The band size was reported using a 50 bp DNA ladder (Takara, Dalian, Liaoning, China) as the reference.

**Table 1:** Primer sequences and characterization for eight microsatellite loci isolated from *Cotinus coggygria*.

Primer	Primer sequence (5’-3’)	Repeat motif	*T*a (°C )	Allele Size (bp)	*A*	*H*_E_	*H*_O_	*F*_IS_	GenBank Accession No.
Cc004	F:CCCCATTAGCTCACTCTCCAR:CGTTCCTATGGGTTCCTCAA	(CTC)7	52	350–381	3	0.301	0.333	−0.114	KJ398949
Cc012	F:AGCAGTGAAGAGCCAACTCCR:AGCTTGCAAAGAATGGGGTA	(TA)7	56	347–363	3	0.420	0.500	−0.200	KJ398957
Cc024	F:GCCCCACCAAGCAATAAAATR:TTCATGGCTCCTCCTTCTTC	(AT)6	56	399–409	4	0.685	0.667	0.028	KJ398969
Cc025	F:CCAAACACCTTTGGGTCTGTR:GGGGAAATGAAACAAGGGTT	(TCT)6	54	123–147	5	0.651	0.667	−0.026	KJ398970
Cc026	F:TGCGCAAACTAATCCAAACAR:TTTCCGCAATAACACACCAA	(ATTT)5	52	246–266	2	0.290	0.333	−0.158	KJ398971
Cc032	F:GGATCAATTTGACCATCATATTCCR:CGGGATTGAGATTGGATTGT	(AT)6	50	333–353	3	0.518	0.750	−0.478	KJ398977
Cc040	F:ACCCTAACCCAAACCCAAACR:ATCGGAAACAACCTCCCTTT	(GAG)6	58	210–216	3	0.163	0.167	−0.023	KJ398985
Cc047	F:AATATCATCCTCCAGCGACGR:TGTTCAGATTCACGGCTGAG	(TTC)9	52	224–242	5	0.743	0.583	0.222	KJ398992

*Ta* PCR annealing temperature; *A* number of alleles; *H*_E_ expected heterozygosity; *H*_O_ observed heterozygosity; *F*_IS_ inbreeding coefficient.

**Table 2:** Genetic diversity parameters for two populations of *Cotinus coggygria*.

Primer	Wuzhi Mountain ( *N* = 6)				Tianlong Mountain ( *N* = 6)			
	*A*	*H*_E_	*H*_O_	*F*_IS_	*A*	*H*_E_	*H*_O_	*F*_IS_
Cc004	2	0.303	0.333	−0.111	3	0.318	0.333	−0.053
Cc012	3	0.439	0.500	−0.154	3	0.439	0.500	−0.154
Cc024	4	0.803	0.500	0.400	2	0.530	0.833	−0.667
Cc025	3	0.537	0.333	0.407	5	0.788	1.000	−0.304
Cc026	2	0.409	0.500	−0.250	2	0.167	0.167	_
Cc032	2	0.530	0.833	−0.667	3	0.530	0.667	−0.290
Cc040	3	0.318	0.333	−0.053	1	0	0	_
Cc047	2	0.409	0.500	−0.250	4	0.742	0.667	0.111

*N* Number of individuals tested; *A* number of alleles; *H*_E_ expected heterozygosity; *H*_O_ observed heterozygosity; *F*_IS_ inbreeding coefficient.

### 3.3. Data Analysis

Preliminary population genetic analyses, including the allele number, the observed/expected heterozygosities, the inbreeding coefficient and linkage equilibrium between markers were performed using GENEPOP version 4.2 [21,22]. Significance testing of the inbreeding coefficient at all loci was performed using FSTAT 2.9.3.2 [23]. The Hardy-Weinberg equilibrium of markers/populations was tested using Fisher’s exact test and with the Markov chain method using Arlequin v3.5 [24].

## 4. Conclusions

In our study, a total of 349 microsatellite loci were isolated. Among the identified SSR loci, 50 microsatellite primer pairs were selected to test the primer amplification efficiency. Of these 50 microsatellite markers, eight primer pairs yielded polymorphic and single locus amplification products. This is the first set of microsatellite markers developed for *Cotinus* and will be useful for assessing the genetic diversity, understanding the population structure, and phylogeographical and landscape genetic patterns of *C. coggygria*.
